# Genotypic Analysis of Meningococcal Factor H-Binding Protein from Non-Culture Clinical Specimens

**DOI:** 10.1371/journal.pone.0089921

**Published:** 2014-02-24

**Authors:** Stephen A. Clark, Jay Lucidarme, Lynne S. Newbold, Ray Borrow

**Affiliations:** Manchester Medical Microbiology Partnership, Public Health England, Clinical Sciences Buildings, Manchester Royal Infirmary, Manchester, United Kingdom; Naval Research Laboratory, United States of America

## Abstract

Factor H-Binding Protein (fHbp) is an outer membrane protein antigen included in two novel meningococcal group B vaccines and, as such, is an important typing target. Approximately 50% of meningococcal disease cases in England and Wales are confirmed using real-time PCR on non-culture clinical specimens only. Protocols for typing fHbp from this subset of cases have not yet been established. Here we present a nested PCR-based assay designed to amplify and sequence *fHbp* from non-culture clinical specimens. From analytical sensitivity experiments carried out using diluted DNA extracts, an estimated analytical sensitivity limit of 6 fg/µL of DNA (<3 genome copies/µL) was calculated. The sensitivity of the assay was shown to be comparable to the *ctrA*-directed real-time PCR assay currently used to confirm invasive disease diagnoses from submitted clinical specimens. A panel of 96 diverse, patient-matched clinical specimen/isolate pairs from invasive disease cases was used to illustrate the breadth of strain coverage for the assay. All *fHbp* alleles sequenced from the isolates matched those derived from previous whole genome analyses. The first-round PCR primer binding sites are highly conserved, however an exceptional second-round PCR primer site mismatch in one validation isolate prevented amplification. In this case, amplification from the corresponding clinical specimen was achieved, suggesting that the use of a nested PCR procedure may compensate for any minor mismatches in round-two primer sites. The assay was successful at typing 91/96 (94.8%) of the non-culture clinical specimens in this study and exhibits sufficient sensitivity to type *fHbp* from the vast majority of non-culture clinical specimens received by the Meningococcal Reference Unit, Public Health England.

## Introduction

Invasive meningococcal disease (IMD) represents a significant threat to the public health of communities globally. Immunisation against the causative organism, *Neisseria meningitidis*, remains the most effective means of curtailing the prevalence of this deadly disease. Vaccines containing peptide-conjugated capsular polysaccharides have been successful in reducing disease caused by capsular groups A, C, W and Y [1]–[4]. Disease caused by group B meningococci, however, remains an ever present problem and the development of vaccines against this subset has proven to be much more challenging. Structural similarities between group B polysaccharide and human foetal antigens inhibit the immunogenicity of polysaccharide-based group-B vaccines [5]. Furthermore, formulations based upon group B outer membrane vesicles (OMVs) are yet to offer expansive immune protection due to immunodominance of the heterogeneous Porin A (PorA) antigen [6]. In a change of approach, two group B vaccines containing subcapsular, outer membrane protein antigens have recently been developed and currently represent the most promising strategy for preventing diverse group B disease [7]. Factor H-Binding Protein (fHbp) is an antigen included in both of these vaccines.

FHbp (previously known as LP2086 and GNA1870) is a surface-exposed lipoprotein composed of two domains (a β-sheet and a β-barrel) with an N-terminal flexible chain that anchors it to the outer membrane [8], [9]. The antigen was identified over a decade ago by two separate groups and is perhaps one of the most studied subcapsular vaccine candidates [10], [11]. The antigen is found among virtually all invasive isolates and enhances survival *in vivo* through its ability to bind human complement factor H, a down-regulator of the host alternative complement pathway [12].

At the time of writing, alleles coding for over 600 variants of the fHbp peptide have been sequenced and deposited into the dedicated PubMLST database (http://pubmlst.org/neisseria/fHbp) [13]. These peptide variants can be categorised using two distinct nomenclatures based upon amino acid sequence identity. One system divides variants into two subfamilies (A and B) [10] and the other into three variant groups (1, 2 and 3) [11]. Subfamily B (sB) corresponds to variant group 1 (v1) whilst subfamily A (sA) encompasses variant groups 2 and 3 (v2 and v3). The PubMLST database (hosted by the University of Oxford, UK) seeks to reconcile these differences by assigning arbitrary, sequential numbers to alleles and peptides as they are submitted. These are then annotated with the corresponding designations from the two systems described above [14]. The PubMLST nomenclature will be primarily referenced within this article.

Whilst considerable amino acid sequence variation occurs in fHbp across invasive populations, several studies have illustrated the cross protective nature of the antigen whereby one fHbp variant can elicit immune protection against strains bearing variants of the same subfamily [8], [10], [11], [15]. This attribute of subfamily-specific cross protection is exploited in an investigational recombinant vaccine (rLP2086), developed by Pfizer. The vaccine contains two lipidated fHbp peptide variants, peptide 45 (sA/v3) and peptide 55 (sB/v1), and trials in adults have shown it to elicit serum bactericidal antibodies (SBA) against diverse group B isolates [16], [17]. In a parallel effort, a multi-component group B vaccine, also containing fHbp, has recently been licenced for use in children and adults in the European Union and Australia [18], [19]. The formulation contains one fHbp variant, peptide 1 (sB/v1), as part of a fusion protein among other antigens [20].

The potential use of these vaccines in the UK and elsewhere emphasises the need for comprehensive enhanced surveillance of disease-causing organisms. These activities aid vaccine coverage predictions and will serve to monitor the ongoing impact of the vaccines should they be utilised. In England and Wales, Public Health England’s Meningococcal Reference Unit (PHE, MRU) provides species confirmation and routine characterisation of IMD isolates. Since July 2010, whole genome sequence analysis has been adopted for all invasive isolates received by the MRU. To date, the genomes of over 900 UK isolates (epidemiological years 2010/2011 and 2011/2012) have been sequenced, annotated and made freely available in the Meningitis Research Foundation’s online Meningococcus Genome Library (MGL, http://www.meningitis.org/current-projects/genome). In recent years, however, approximately 50% of IMD cases in England and Wales were confirmed solely by real-time PCR testing of clinical specimens from which no isolate could be obtained [21]. Limiting factors such as low DNA concentration and interference from human DNA have prevented the adoption of whole genome analysis from these ‘non-culture’ specimens.

PCR-based assays designed for Multilocus Sequence Typing (MLST) and typing of PorA loci from clinical specimens have been developed [22], [23]. An assay to characterise fHbp in this way is, however, not currently available. Consequently, the variation of fHbp among this large proportion of clinical cases remains unknown. This represents a significant epidemiological knowledge gap and may influence coverage estimations of fHbp-containing vaccines. In order to complete the picture of fHbp distribution in England and Wales, a PCR-based assay has been developed to sequence *fHbp* from non-culture clinical specimens containing potentially low-copy number meningococcal DNA. This work describes the assay and the activities carried out to illustrate its sensitivity to the target template and its coverage of diverse meningococcal strains.

## Methods

### Ethics Statement

As this study involved the use of anonymised specimens that were surplus to clinical need, ethical approval was not required in accordance with National Research Ethics Service (NRES) guidelines.

### Isolates and Clinical Specimens

#### Analytical sensitivity

Seven invasive meningococcal isolates, representing the predominant clonal complexes and fHbp variant groups, were selected to assess the analytical sensitivity of the assay (Table S1).

#### Validation panel

A validation panel comprising 96 clinical isolates was compiled from disease cases confirmed by both organism isolation and PCR on a clinical sample (2010–2012). All isolates selected have previously undergone whole genome analysis and are included in the MGL. Each isolate was paired with its corresponding patient-matched clinical specimen. Selection of the isolate/specimen pairs was based upon MLST and fHbp data of the cultured isolate. The panel was selected to represent diverse clonal complexes (CC), sequence types (ST) and fHbp variants, approximating the distribution of CCs within England and Wales (Table S1). The diversity of these specimens was confirmed by mapping the respective STs against their corresponding CCs as determined by eBURST V3 analysis [24] of the entire MLST dataset (PubMLST database, 30/12/2013, Fig. S1). To improve the resolution of the ST-11 CC, ribosomal MLST (rMLST) and SplitsTree4 analysis (www.splitstree.org) was performed on available ST-11 CC genomes, against which the selected panel isolates were mapped [25].

#### Neisseria lactamica isolates

In addition to the validation panel, a selection of six diverse *N. lactamica* isolates was made based upon the distribution of the STs within the known population of the organism [26]. Isolates from distinct STs and CCs were chosen (Table S1). Primer complementarity was assessed using an alignment of sequences from 16 *N. lactamica* isolates submitted to the PubMLST database (Table S2).

### DNA Extraction

All DNA extractions were performed using the Qiagen DNeasy Blood and Tissue Kit.

DNA extraction from clinical specimens was carried out in accordance with Qiagen DNeasy Blood and Tissue Handbook (July 2006). A volume of 100 µL of each clinical specimen was used where possible. If less than 100 µL was available, the maximum volume available was used and the remainder was made up using phosphate-buffered saline (PBS). Extracts from clinical specimens were eluted twice using 50 µL buffer AE per elution.

DNA extraction from cultured isolates was adapted from the manufacturer’s gram-negative bacteria protocol (Qiagen Blood and Tissue Handbook, July 2006). All isolate extracts were eluted in 2×75 µL buffer AE. *Neisseria lactamica* isolate extracts prepared previously were used [26]. All DNA extracts were stored at 4°C.

### Analytical Sensitivity

Extracted DNA from each isolate was quantified using Qubit 2.0 fluorometer (Life Technologies, USA) following the manufacturers’ protocol and adjusted to 6 ng/µL. A series of eight 10-fold dilutions was carried out to an estimated DNA concentration of 60 ag/µL (Table 1).

**Table 1:** Serial dilutions of meningococcal extracts, corresponding estimated DNA concentration and calculated genome copy number (estimate based upon a 2.2 Mbp genome).

Dilution	DNA Concentration (per µL)	Calculated Single Genome Copies (per µL)
Starting Conc.	6 ng	2.53×10^6^
10^−1^	600 pg	2.53×10^5^
10^−2^	60 pg	2.53×10^4^
10^−3^	6 pg	2530
10^−4^	600 fg	253
10^−5^	60 fg	25.3
10^−6^	6 fg	2.53
10^−7^	600 ag	0.253
10^−8^	60 ag	0.0253

Semi-quantitative cycle threshold (Ct) values were obtained using the current in-house *ctrA*-directed TaqMan assay, which is an adaptation of a previously published protocol [27]. The assay was performed using the ABI 7500 Fast Real-Time PCR System (Life Technologies, USA) and features cyanine dye (Cy5) probes targeting sequences within the *ctrA* capsule synthesis gene. TaqMan assay plates with pre-lyophilised primer/probes (Life Technologies, USA) were reconstituted in molecular grade water before 5 µL of the diluted DNA extracts was added. The Ct value is given as the cycle-number at which the fluorescent signal meets a calculated background threshold, confirming the presence and amplification of the target sequence. A Ct value of >45 is currently reported as negative.

### Finalised Assay Protocol

Detailed descriptions of the assay development (including primer design and optimisation) are included as Method S1.

#### Nested PCR

To attain the sensitivity and specificity required for amplification from non-culture specimens, a nested-PCR protocol, utilising two consecutive PCR rounds, was adopted. PCR primers are listed in Table 2. All PCR reagents and volumes are listed in Table 3. Round one PCR reactions were performed in a 50 µL reaction containing 10 µL of extracted template DNA. Round two reactions were performed using a 25 µL reaction containing 2 µL of round one product. Negative controls were prepared by replacing DNA extract with 10 µL of molecular-grade water in the round one reaction. This was then transferred to PCR round two along with experimental products.

**Table 2:** PCR and sequencing primers.

Primer ID	Primer Use (direction)	Sequence (5′ to 3′)	Reference
*1869-2F*	Round One PCR (Fwd)	GAAGAAATCGTCGAAGGCATCAAAC	[26], [29]
*1871Ralt*	Round One PCR (Rev)	ATGCCGATACGCAGTCC(G/C)GTAAAC	[26], [29]
*fHbpRd2F*	Round Two PCR (Fwd)	GTTATGCCAAGGGCGAATTGAACC	n/a
*fHbpRd2R*	Round Two PCR (Rev)	GTGCGGATTTCCGGCAG(G/A)ATCA	n/a
*gna1870F*	Sequencing (Fwd)	TGACCTGCCTCATTGATGC	[30]
*fHbpseqR2*	Sequencing (Rev)	AGGACGGG(G/A)CGGTT(G/A)AAATC	n/a
*gna1870S2*	Sequencing (Fwd)	CAAATCGAAGTGGACGGGCAG	[30]
*gna1870S3*	Sequencing (Rev)	TGTTCGATTTTGCCGTTTCCCTG	[30]

**Table 3:** Standard nested-PCR mastermix reagent volumes.

PCR Round	Reagent	Volume per Reaction (µL)
Round One	10X PCR Buffer	5
	*1869-2F* (5 µM)	5
	*1871Ralt* (5 µM)	5
	DNTP Mix	1
	HotStarTaq	0.25
	Molecular-grade Water	23.75
	DNA Extract	10
Round Two	10X PCR Buffer	2.5
	*fHbpRd2F* (5 µM)	2.5
	*fHbpRd2R* (5 µM)	2.5
	DNTP Mix	0.5
	HotStarTaq	0.125
	Molecular-grade Water	14.875
	Round One Product	2

Both PCR rounds use the same temperature and thermocycling conditions/times. An initial activation step of 95°C for 15 minutes was followed by 35 (round one) or 30 (round two) cycles of denaturation at 95°C for 30 seconds, annealing at 63°C for 30 seconds and extension at 72°C for 80 seconds. These were followed by a final extension step of 72°C for 7 minutes. Following PCR round two, amplification was confirmed by 2% agarose gel electrophoresis.

Successfully amplified products were purified prior to sequence analysis using ExoSap IT PCR product cleanup kit (Affymetrix, USA). The purified product was diluted in molecular grade water based upon visual assessment of gel band intensity. A 1 in 4 dilution was performed for concentrated products exhibiting relatively strong gel bands, whilst products with average intensity gel bands were diluted 1 in 3. Products with weak gel bands were diluted 1 in 2.

### Sanger Sequencing

Sequence analyses were performed using Big Dye Terminator v3.1 cycle sequencing kit (Life Technologies, USA). Big Dye terminator reaction mix was used in a 1/8^th^ reaction according to the manufacturer’s protocol (2002). Products were cleaned by sodium acetate/ethanol precipitation and resuspended in 15 µl of Hi-Di Formamide (Life Technologies, USA) before analysis using an ABI 3130xl Genetic Analyser. Sequence assembly and analysis was carried out using Sequencher v4.7 (Gene Codes Corporation, Ann Arbor, MI USA).

## Results

### Analytical Sensitivity

To assess the sensitivity of the PCR protocol to the target template, DNA was extracted from seven diverse meningococcal isolates and diluted in series to an approximate concentration of 60 ag/µL (0.025 genome copies (GC)/µL) (Table 1). A volume of 5 µL of extract dilution was initially used in a 25 µL round one PCR reaction. PCR products of the appropriate size were visible on the agarose gel after the first PCR round for all extracts down to a DNA concentration of 60 fg/µL (25.3 GC/µL). For PCR round two, varying amounts of round one product (1 µL, 2 µL and 5 µL) were added to a 25 µL final reaction volume. After the second PCR round, detectable amplification occurred in all extracts to a DNA concentration of 6 fg/µL (2.53 GC/µL). At 600 ag/µL (<1 GC/µL), five of the seven extracts produced appropriate amplicons. No amplification was seen at 60 ag/µL. The volume of round one product transferred into the round two reactions determined the intensity of the resultant bands, however, increasing the volume did not induce amplification in otherwise PCR-negative extracts. Using 2 µL of round one product was therefore deemed sufficient and this volume was used for all subsequent reactions.

**Figure 1:**
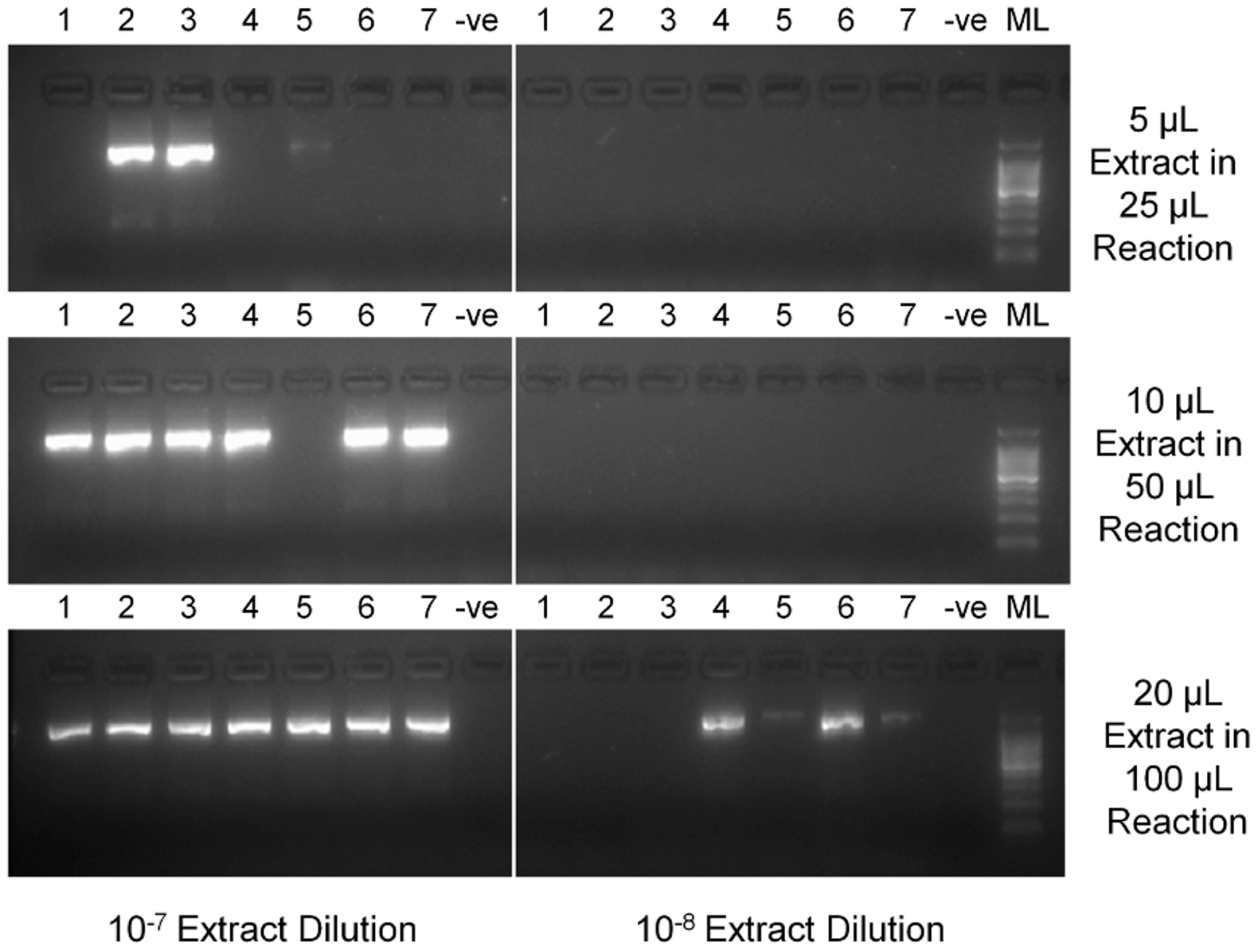
Gel image of amplicons produced using varying extract volumes. Annotated gel image of round two PCR products from nested PCR using varying volumes of diluted DNA extracts in the preceding round one reaction. The products for all seven isolate extracts at the highest two dilutions are shown. Wells to the left of the image contain 10^−7^ dilution extracts (600 ag/µL) and wells adjacent to the molecular weight ladder contain 10^−8^ dilution extracts (60 ag/µL). The extract/reaction volumes (µL) are indicated on the right of the image. Extract numbers refer to the order in which the isolates are listed in Table S1. ML = Molecular ladder.

To determine the influence of DNA extract volume in the round one reaction, three extract volumes of the two highest dilutions (10^−7^ and 10^−8^) were added to round one reactions. The proportion of extract used in each reaction was kept constant: 5 µL/25 µL, 10 µL/50 µL and 20 µL/100 µL. The amount of extract used in round one was shown be an important factor in the likelihood of gaining positive amplification. Figure 1 shows the round two products obtained following the respective round one reactions. The 600 ag/µL (10^−7^) extracts from all seven isolates produced appropriate amplicons when 20 µL of the diluted extract was used for the first round. Amplification occurred from only six and three of the seven extracts when 10 µL and 5 µL of extract were used, respectively. PCR products were visible for four of the seven isolates when 20 µL of the 60 ag/µL (10^−8^) dilution was used. No amplification was seen in the other reactions at this dilution.

In order to compare the DNA concentrations of these dilutions to routinely submitted specimens, the dilutions were applied to the in-house *ctrA*-directed TaqMan real-time PCR assay. As expected, Ct values of the extracts steadily increased as the DNA concentration within each dilution decreased (Table 4). An average increase of approximately 4 cycles was observed for each successive extract dilution. Each extract was tested once up to 6 fg/µL (10^−6^). For dilutions of 10^−7^ and 10^−8^, the assay was repeated three times to counter any of the stochastic sampling effects observed previously. At 6 fg/µL (the nested-PCR analytical sensitivity limit) an average Ct value of 37.86 was obtained. At 10^−7^, only one of the extracts produced a positive result and achieved a Ct value of 41. No positive Ct value was obtained for any extract at the lowest dilution (10^−8^).

**Table 4:** Ct values generated using real-time TaqMan PCR assay.

Isolate	Ct Value per Extract Dilution											
	10^−1^	10^−2^	10^−3^	10^−4^	10^−5^	10^−6^	10^−7^a	10^−7^b	10^−7^c	10^−8^a	10^−8^b	10^−8^c
M08 0240297	16.8	20.1	25.0	28.3	32.1	36.3	41.0	−ve	−ve	−ve	−ve	−ve
M07 0240954	17.4	20.7	25.3	28.9	32.3	38.7	−ve	−ve	−ve	−ve	−ve	−ve
M07 0241036	20.6	25.8	29.0	33.0	38.1	41.0	−ve	−ve	−ve	−ve	−ve	−ve
M07 0241073	17.0	20.8	25.4	28.7	32.9	38.5	−ve	−ve	−ve	−ve	−ve	−ve
M07 0240725	17.4	22.0	25.2	−ve	32.3	36.1	−ve	−ve	−ve	−ve	−ve	−ve
M08 0240113	17.5	22.1	25.3	27.6	32.2	36.5	−ve	−ve	−ve	−ve	−ve	−ve
M08 0240032	17.2	21.8	25.3	28.4	32.0	37.9	−ve	−ve	−ve	−ve	−ve	−ve
Neg. Control	−ve	−ve	−ve	−ve	−ve	−ve	−ve	−ve	−ve	−ve	−ve	−ve
Mean *CT*	17.70	21.85	25.79	29.15	33.13	37.86	41.00	n/a	n/a	n/a	n/a	n/a

### Validation Panel

A total of 96 diverse meningococcal isolates with corresponding non-culture clinical specimens were selected. DNA extracts from the clinical specimens were tested using the finalised nested-PCR protocol, however, the isolate extracts were tested using both PCR rounds independently.

**Figure 2:**
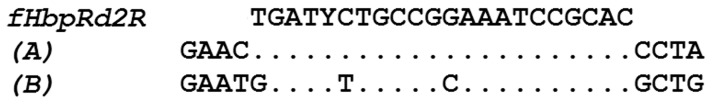
Conservation of the *fHbpRd2R* primer binding site. Single dots denote bases matching that of the PCR primer (Top sequence, 3′–5′ direction). Sequence A is representative of 438/443 (98.8%) meningococcal isolates aligned (Table S2). Sequence B features the *fHbpRd2R* primer site mismatch. This was found in two isolates within the MGL (0.22%) and 25% of *N. lactamica* sequences aligned.

For PCR round one, all 96 isolates produced an appropriate amplicon. During PCR round two, all but one isolate produced a PCR product (Table S3). Three attempts failed to produce an amplicon for isolate M11 240189 for PCR round two. To determine primer site conservation, the *fHbp* allele sequence plus flanking regions for this isolate were aligned against the second-round PCR primers. Three base mismatches were found within the *fHbpRd2R* binding site (Figure 2), one of which was located at the 3′ end from which extension occurs. Following a BLAST search of the 923 isolates within the MGL data, only one other isolate (M11 240984) was found to feature these mismatches within this primer binding site (together representing 0.22% of MGL isolates). Sequencing of the 95 successfully amplified products revealed *fHbp* alleles matching the alleles assigned in the MGL from whole genomic data. M11 240402 is an *fHbp* null isolate; no sequencing trace was obtained for this product.

For the clinical specimens, 83 of the 96 (86.5%) produced an appropriate PCR product using the nested PCR procedure on the first attempt (Run 1, Table S3). PCR negative extracts were repeated in two subsequent runs yielding six further products (Runs 2 and 3, Table S3). The seven remaining PCR-negative extracts were tested at an increased extract/reaction volume (20 µL/100 µL) and PCR cycle numbers (round one increased by 10 cycles to x45, round two increased by 5 cycles to x35, Run 4, Table S3). Five of these seven extracts still failed to amplify. These specimens were considered PCR negative and no further attempt at amplification was performed. After four attempts, therefore, 91 of the 96 of the clinical specimens yielded PCR products (94.8%). Curiously, the specimen corresponding to isolate M11 240189 (featuring *fHbpRd2R* site mismatch) amplified successfully using the nested PCR procedure. This may indicate that primer site mismatches can be overcome by using a preceding PCR round.

All but two of the amplified products from specimens contained *fHbp* alleles matching those of their corresponding isolates. The alleles of the two incongruent specimens, M11 922854 and M11 915746, both differed from their corresponding isolates’ alleles by one base resulting in alleles not assigned in the PubMLST database. Re-amplification and sequencing of the specimens was performed to account for PCR copying errors that may have occurred. The resulting alleles matched the corresponding isolates and confirmed that errors were likely to have occurred early in the PCR cycle repeats.

### Neisseria Lactamica Isolates

To provide insight into the conservation of the PCR primer sites beyond the species level, DNA extracts from 6 diverse *N. lactamica* isolates [26] were tested in each PCR round individually (Table S1). Conservation of the primer site sequences in a related species could be indicative of functional constraint of the peptides for which they code and would increase confidence in the longevity of the primers. Recent work has confirmed that *N. lactamica* do not possess *fHbp*, and that the allele for NLA18150 (a putative opacity protein) can be found in its place at this locus flanked by genes common to both species [26], [28]. The PCR primers, targeting sequences within these flanking genes, should however produce an amplicon if the binding sites are conserved. For PCR round one, amplification occurred from all 6 *N. lactamica* isolates. For PCR round two, however, amplification was only observed in 2 of the 6 isolates.

In order to determine the cause of the negative results, DNA sequences of NLA18150 and the flanking regions from 16 *N. lactamica* genomes were downloaded from the PubMLST database and aligned with the PCR primer sequences. The genomic sequences were well conserved for all primer sites apart from *fHbpRd2R*. At this site, 4 of the 16 *N. lactamica* sequences contained the identical primer mismatches observed in the meningococcal validation isolate M11 240189 (Figure 2). Five of the remaining sequences contained a single base substitution at position 9 of the primer (3′-5′), however this is unlikely to affect amplification.

To further assess the potential ability of the nested PCR design to compensate for round two primer site polymorphisms, 2 µL of round one product from all 6 *N. lactamica* isolates was added to 25 µL round two reactions. The use of round one product as the template for the round two reaction appears to have compensated for the mismatch as PCR products of appropriate size were obtained for all extracts.

## Discussion

For the purposes of enhanced surveillance, the genotyping of meningococcal Factor H-Binding Protein (fHbp) from non-culture clinical specimens is required. To this end, this study describes the development and validation of a sensitive PCR-based assay to aid vaccine coverage predictions and provide ongoing epidemiological surveillance data.

To measure the sensitivity of the PCR assay, analytical sensitivity experiments were performed using diluted DNA extracts from meningococcal isolates. The use of cultured isolates allowed for direct quantification of pure meningococcal DNA. In contrast, the use of clinical specimens may have been complicated by the presence of human DNA within the extracts. For the nested PCR protocol, an analytical sensitivity limit of 6 fg/µL of DNA (<3 genome copies/µL) was calculated when using 5 µL of DNA extract in the PCR round-one reaction. At lower DNA concentrations, stochastic DNA sampling effects come into play and the likelihood of obtaining an amplified product becomes dependent on the volume of extract used in the round one reaction. This is illustrated in Figure 1, in which the number of extracts producing amplicons increases as the round one DNA extract volume used is increased. Based on these data, the assay’s sensitivity can be increased by a factor of 10 (to 600 ag/µL) by using 20 µL of extract in the first PCR round (in a 100 µL reaction). Whilst maximum sensitivity is desired, the assay is to be used routinely and the adoption of a 100 µL round one reaction per specimen would be impractical not only in terms of use of consumables but, more importantly, in the consumption of the valuable DNA extract. A 10 µL/50 µL reaction volume would be more appropriate and, based on these data, should amplify the majority of specimens containing a DNA concentration of ≥600 ag/µL. It is recommended that this reaction volume be used in the first instance. Increasing the extract volume is of course one of several parameters that could be amended in order to gain a product from specimens that prove difficult to amplify. These also include increasing the PCR cycle number. Although the assay has been designed to use as a low cycle number as possible to prevent the production of non-specific amplicons, increasing the first round cycle number could provide additional sensitivity if required. Furthermore, increasing the volume of round one product added to the second PCR round reaction has been shown to increase the final PCR product concentration.

The dilution series was tested using the *ctrA*-directed TaqMan real-time PCR assay used for routine case confirmation. The results were comparable to those of the nested-PCR analytical sensitivity, indicating that, using this assay, amplification of *fHbp* should be possible for all specimens with meningococcal DNA concentrations of ≥6 fg/µL. This corresponds to an approximate Ct value of 38 in this TaqMan assay. Of the non-culture specimens received by the MRU in 2011–2012, approximately 94% achieved a Ct value of ≤38. This proportion of clinical specimens should therefore yield a PCR product when using at least 5 µL of extract in PCR round one. By increasing the PCR round one extract volume to 20 µL, this predicted proportion could be increased to 98% (<Ct41).

The ability of the assay to amplify *fHbp* from the widest possible array of meningococcal specimens is of utmost importance. The conservation of the primer binding sequences was assessed by testing the assay against a large panel of meningococci, representative of major disease-causing clonal populations in the UK and other westernised countries. By pairing 96 clinical specimens with cultured meningococcal isolates from the same disease cases, the results attained could be compared to the data produced using whole genome analysis of the isolates to confirm the accuracy of the assay and identify any discrepant results.

PCR products were obtained from 86.5% of the clinical specimens on the first attempt. After repeated testing with the nested PCR protocol, a total of 91 of the 96 specimen extracts yielded products, highlighting the benefit of repeat testing of unamplified extracts and making amendments to the PCR parameters, when necessary. For the 96 corresponding meningococcal isolates, the round one and two PCR protocols were tested individually. During PCR round two, one of the isolate extracts failed to amplify. This was later found to be due to mismatches in the reverse primer binding site. This isolate appears to be one of two isolates within the MGL with this identical discordant sequence at this site (Figure 2). The two isolates are not of the same clonal complex (ST-282 CC and ST-32 CC) and the polymorphic site is within the flanking gene (NMB1871), suggesting it is simply a rare variation at this site. Interestingly, the extract from the clinical specimen matching this validation isolate (M11 909694) was successfully amplified after both nested PCR rounds. Subsequent sequencing of this amplicon showed that the *fHbp* allele matches that of the corresponding isolate (allele 69). This is a rare allele in the UK and it is associated with the similarly rare ST-282 complex, that of the isolate. The *fHbp* allele match is therefore unlikely to have resulted from cross contamination from another specimen/extract. In this case, the increase in the template DNA concentration during PCR round one may have improved the likelihood of semi-complementary primer binding in the second round. Early round two products would therefore feature the exact *fHbpRd2R* primer site, and would more efficiently amplify from that point. If this is indeed the case, the use of a nested PCR procedure may compensate for this rare second round primer mismatch and perhaps other primer disparities that may be encountered.

The sequencing results illustrate the conservation of the sequencing primers to sites flanking/within a range of *fHbp* alleles. For the isolates, all amplicons contained *fHbp* alleles matching those assigned from previously acquired whole genome data. All of the PCR products from the specimens were sequenced successfully and matched their corresponding isolate in terms of *fHbp* allele. Initially, two specimens (M11 922854 & M11 915746) produced alleles differing from the matched isolate by one nucleotide base, however, repeated PCR and sequencing of the extracts resolved the discrepancies. The resulting *fHbp* alleles matched those of the respective isolates suggesting PCR copying errors had initially occurred. Copying errors were only observed in the clinical specimen subset indicating that the low quantity of DNA may be increasing the PCR copying error-rate. Copying errors that occur during the early PCR cycles may be represented to a significant degree in the sequence traces of extracts of low starting template number. The future adoption of high fidelity ‘proof reading’ polymerase enzymes may be warranted for sequencing of clinical specimens. In light of these results, retesting of specimens producing *fHbp* alleles that are not assigned in the PubMLST database may also be prudent.

hAn assessment of primer binding site conservation beyond the species level was made applying the assay to isolates of *N. lactamica*. The closely related commensal does not possess *fHbp* however both sets of PCR primers target neighbouring genes that are common to both species, therefore, amplification should occur if the sites are sufficiently conserved. The round one PCR primers amplified the locus in all six isolates tested. These findings are reflected in a previous study in which the same round one primers (*1869-2F* and *1871Ralt*) were used to confirm the absence of *fHbp* in an additional 44 *N. lactamica* isolates [26]. The round two PCR primer sites were, however, less conserved and the identical *fHbpRd2R* primer mismatch seen in two meningococcal isolates (Fig. 2) was shown to be present in 25% of the *N. lactamica* genomes aligned (Table S2). This finding suggests that the presence of this polymorphism in a tiny proportion of meningococcal isolates may be a result of recombination with *N. lactamica.* Amplification of *fHbp* from all 6 *N. lactamica* isolates was achieved when using a nested PCR method, providing more evidence to support the suggestion that the nested-PCR can compensate for round two primer site discrepancies.

To conclude, this assay has the sensitivity to type *fHbp* from the vast majority of non-culture IMD specimens from England and Wales. The development of this assay provides an opportunity to complete the epidemiological picture of this important vaccine antigen.

## Supporting Information

Figure S1 eBURST and SplitsTree diagrams illustrating the distribution of the validation panel isolates among the known meningococcal population diversity. eBURST analysis was performed using all MLST allelic profile data obtained from the PubMLST database on 30/12/2013. Briefly, the eBURST program divides STs into groups based on the relatedness of the allelic variants at the seven MLST loci. For this analysis, the most stringent level was used in which a minimum of 6 identical loci is required for group definition. Single locus variants (SLVs, i.e. STs that differ at only one of the seven MLST loci) are grouped together around a ‘founder ST’, which possesses the most related SLVs. These are predicted to represent an ancestral ST from which all other ST variants were derived. Each group roughly correlates with established clonal complexes in which constituent STs have ≥4 loci in common with the ancestral ST. The eBURSTs are annotated with the STs of the validation panel isolates (highlighted in red) to illustrate their genetic diversity. The eBURSTs roughly correlate with 7 predominant clonal complexes: ST-41/44 complex (A), ST-269 complex (B), ST-32 complex (C), ST-22 and ST-23 complexes (D), ST-213 complex (E) and ST-60 complex (F). ST-10281 is a member of ST-213 complex, however, in this analysis, there is no SLV linking ST-10281 to a member of this eBURST group. For the ST-11 complex, four of the six validation panel isolates were of ST-11. The higher resolution provided by ribosomal MLST (rMLST) was utilised to produce a SplitsTree diagram illustrating the rMLST profile of all ST-11 complex genomes within the PubMLST database (n = 177). The isolates used in the assay validation were then highlighted to illustrate the diversity.
(PDF)

Table S1 *N. meningitidis* and *N. lactamica* validation isolates and clinical specimens.
(PDF)

Table S2 Isolates from which *fHbp* and flanking sequences were used to assess primer site conservation and identify putative primer candidates.
(PDF)

Table S3 Results of PCR and sequencing of validation panel isolate/specimen pairs.
(PDF)

Method S1 Assay Development.
(PDF)
