# Supplementary material for: Genotypic Analysis of Meningococcal Factor H-Binding Protein from Non-Culture Clinical Specimens
Source: PLoS One. 2014 Feb 24;9(2):e89921. doi: 10.1371/journal.pone.0089921 (PMC3933679; doi:10.1371/journal.pone.0089921)
Supplement: Figure S1 — eBURST and SplitsTree diagrams illustrating the distribution of the validation panel isolates among the known meningococcal population diversity. eBURST analysis was performed using all MLST allelic profile data obtained from the PubMLST database on 30/12/2013. Briefly, the eBURST program divides STs into groups based on the relatedness of the allelic variants at the seven MLST loci. For this analysis, the most stringent level was used in which a minimum of 6 identical loci is required for group definition. Single locus variants (SLVs, i.e. STs that differ at only one of the seven MLST loci) are grouped together around a ‘founder ST’, which possesses the most related SLVs. These are predicted to represent an ancestral ST from which all other ST variants were derived. Each group roughly correlates with established clonal complexes in which constituent STs have ≥4 loci in common with the ancestral ST. The eBURSTs are annotated with the STs of the validation panel isolates (highlighted in red) to illustrate their genetic diversity. The eBURSTs roughly correlate with 7 predominant clonal complexes: ST-41/44 complex (A), ST-269 complex (B), ST-32 complex (C), ST-22 and ST-23 complexes (D), ST-213 complex (E) and ST-60 complex (F). ST-10281 is a member of ST-213 complex, however, in this analysis, there is no SLV linking ST-10281 to a member of this eBURST group. For the ST-11 complex, four of the six validation panel isolates were of ST-11. The higher resolution provided by ribosomal MLST (rMLST) was utilised to produce a SplitsTree diagram illustrating the rMLST profile of all ST-11 complex genomes within the PubMLST database (n = 177). The isolates used in the assay validation were then highlighted to illustrate the diversity. (PDF) [file pone.0089921.s001.pdf]

A:

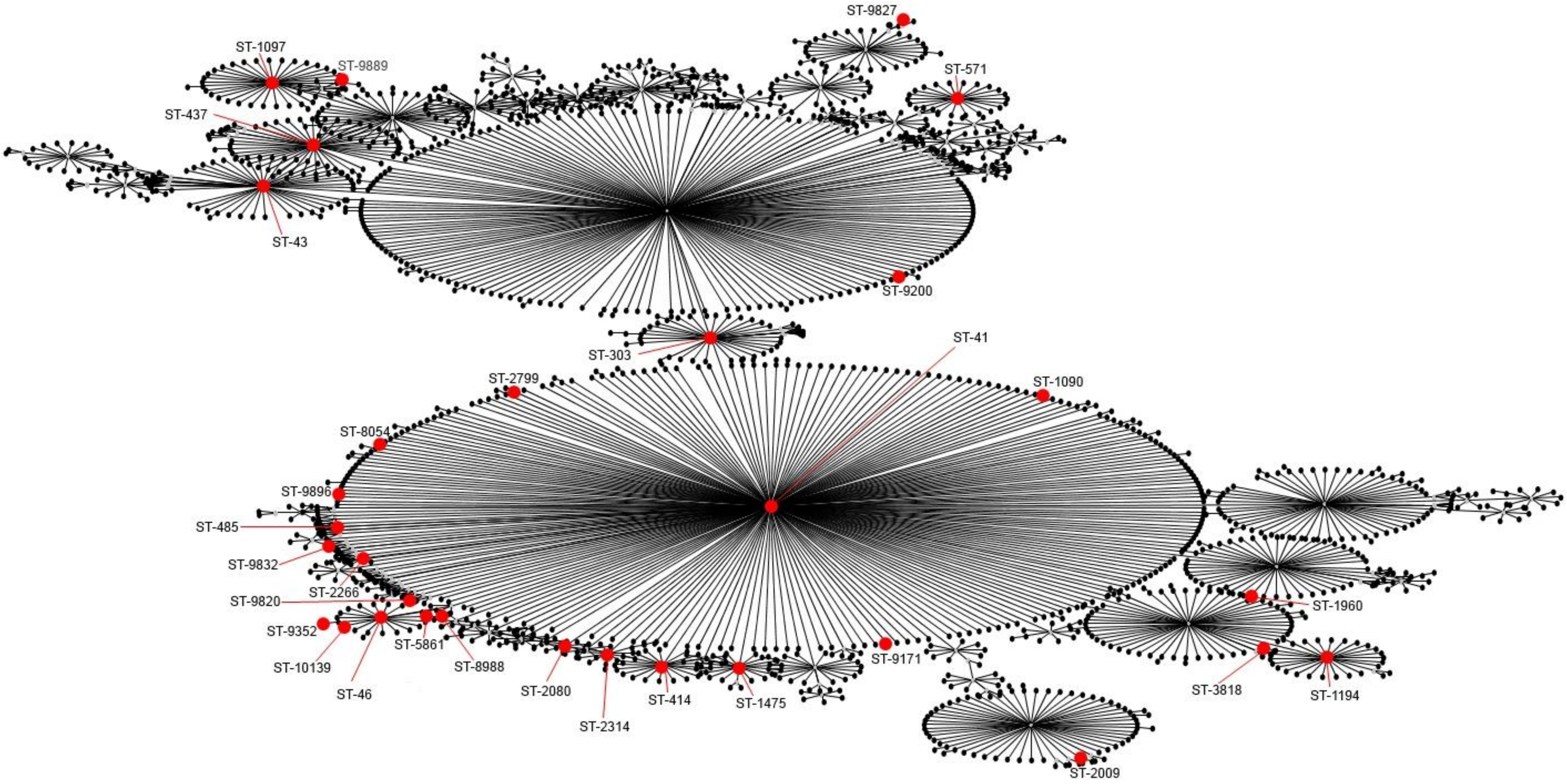

ST-9827
ST-1097
ST-9889
ST-571
ST-437
ST-43
ST-9200
ST-41
ST-303
ST-2799
ST-1090
ST-8054
ST-9896
ST-485
ST-9832
ST-2266
ST-9820
ST-9352
ST-5861
ST-8988
ST-10139
ST-46
ST-2080
ST-2314
ST-414
ST-1475
ST-9171
ST-1960
ST-3818
ST-1194
ST-2009

B:

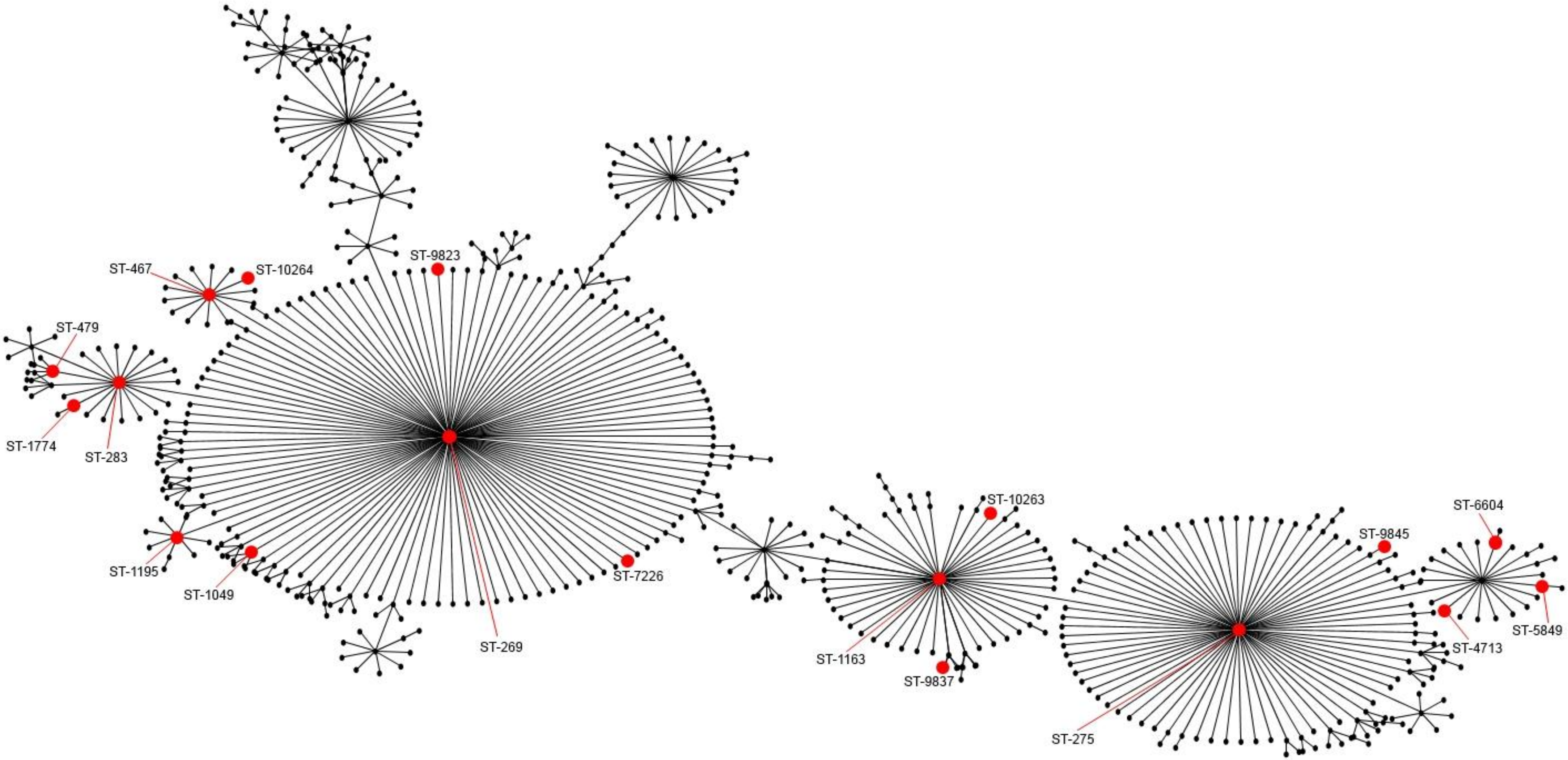

ST-9823
ST-467
ST-10264
ST-479
ST-1774
ST-283
ST-1195
ST-1049
ST-7226
ST-269
ST-10263
ST-1163
ST-9837
ST-6604
ST-9845
ST-5849
ST-4713
ST-275

C:

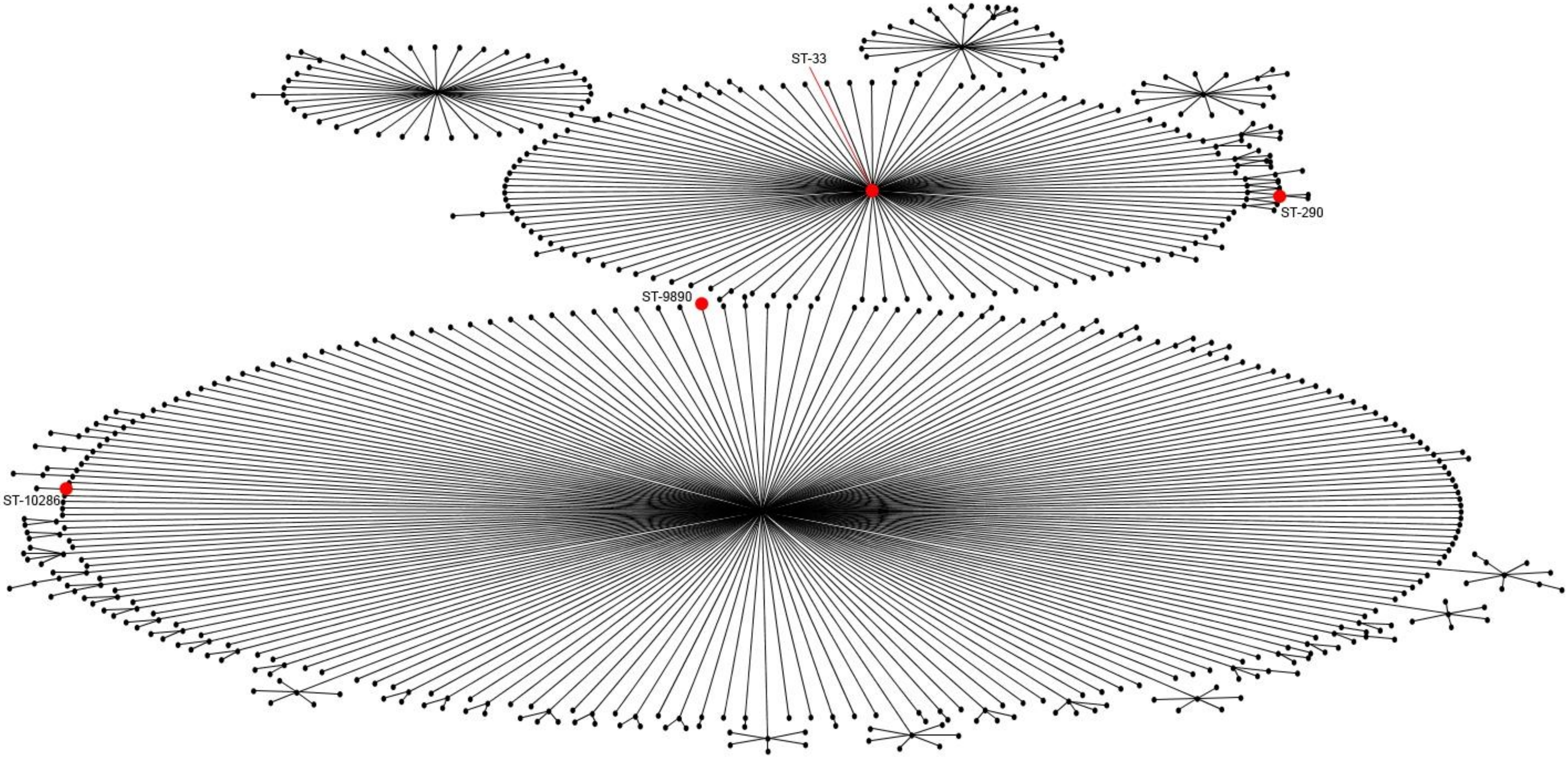

D:

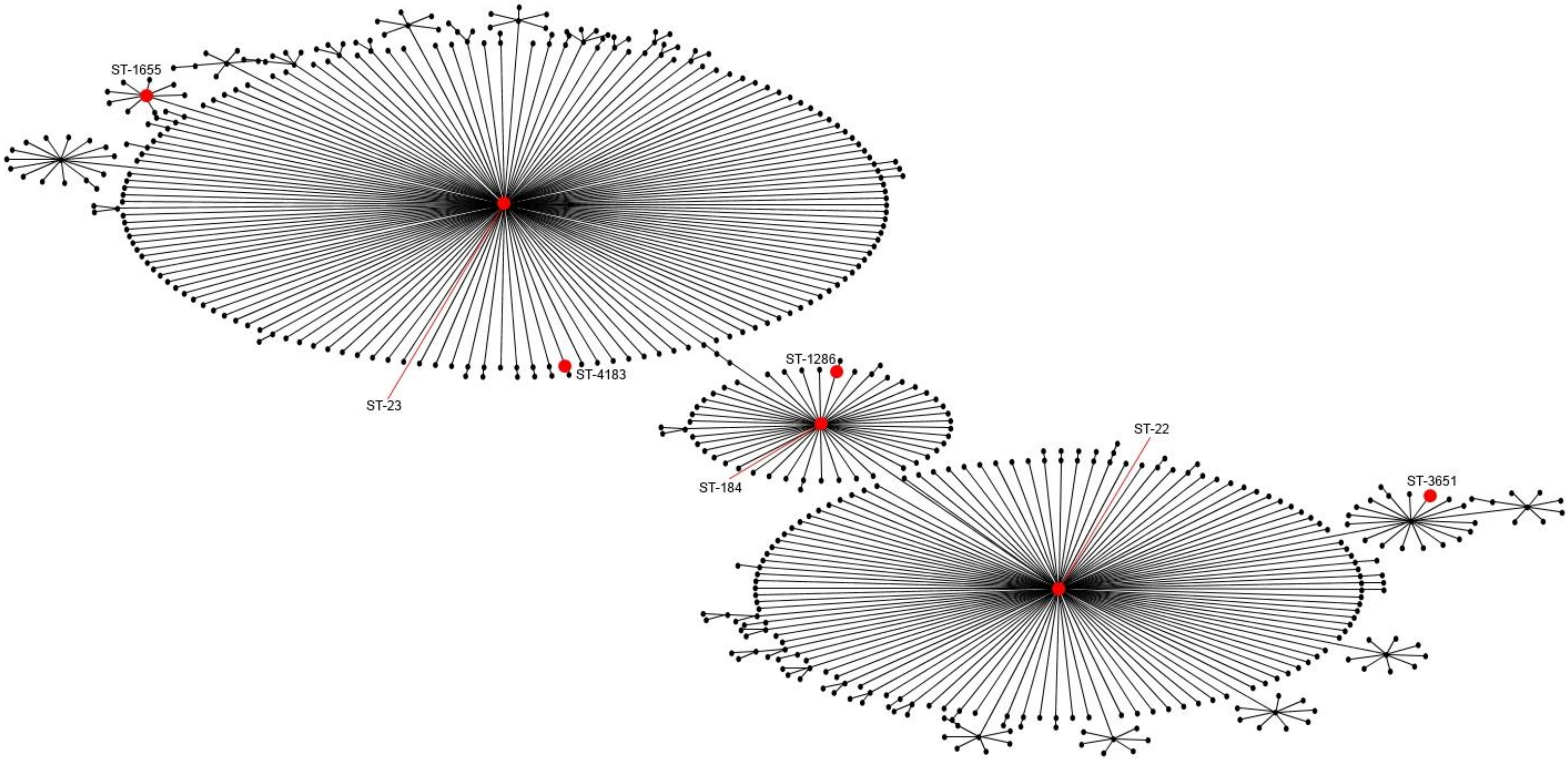

E:

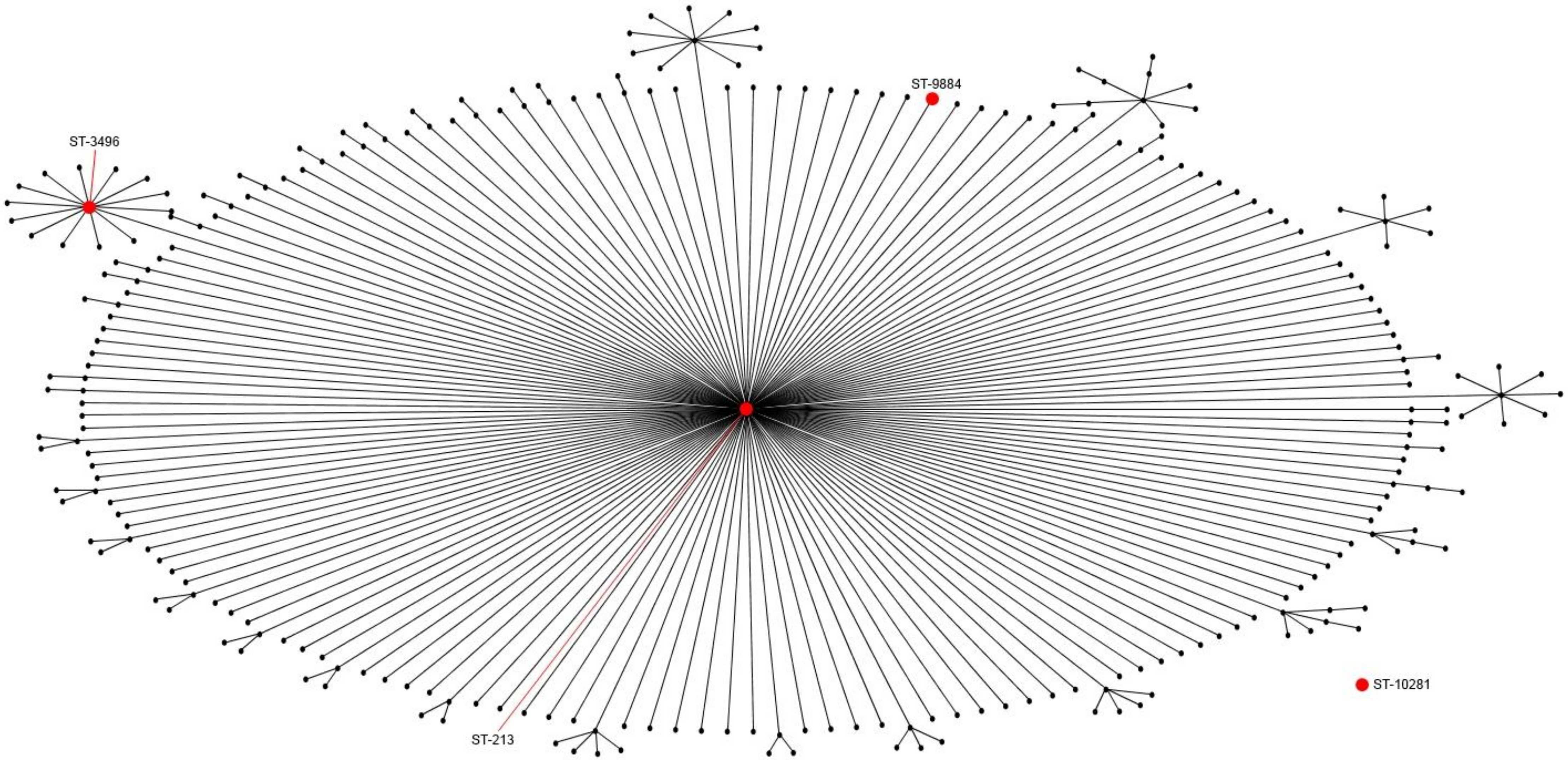

F:

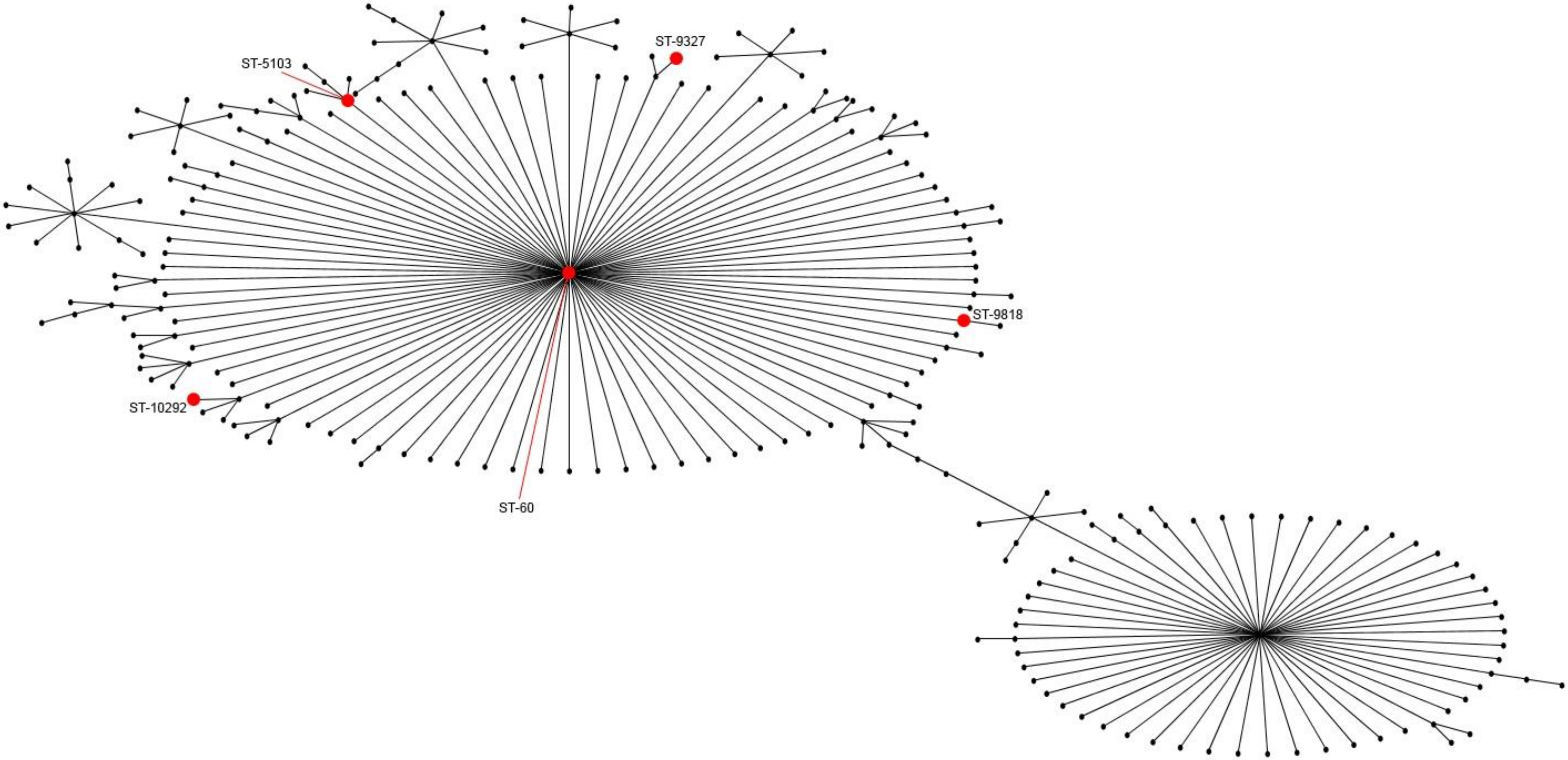

G:

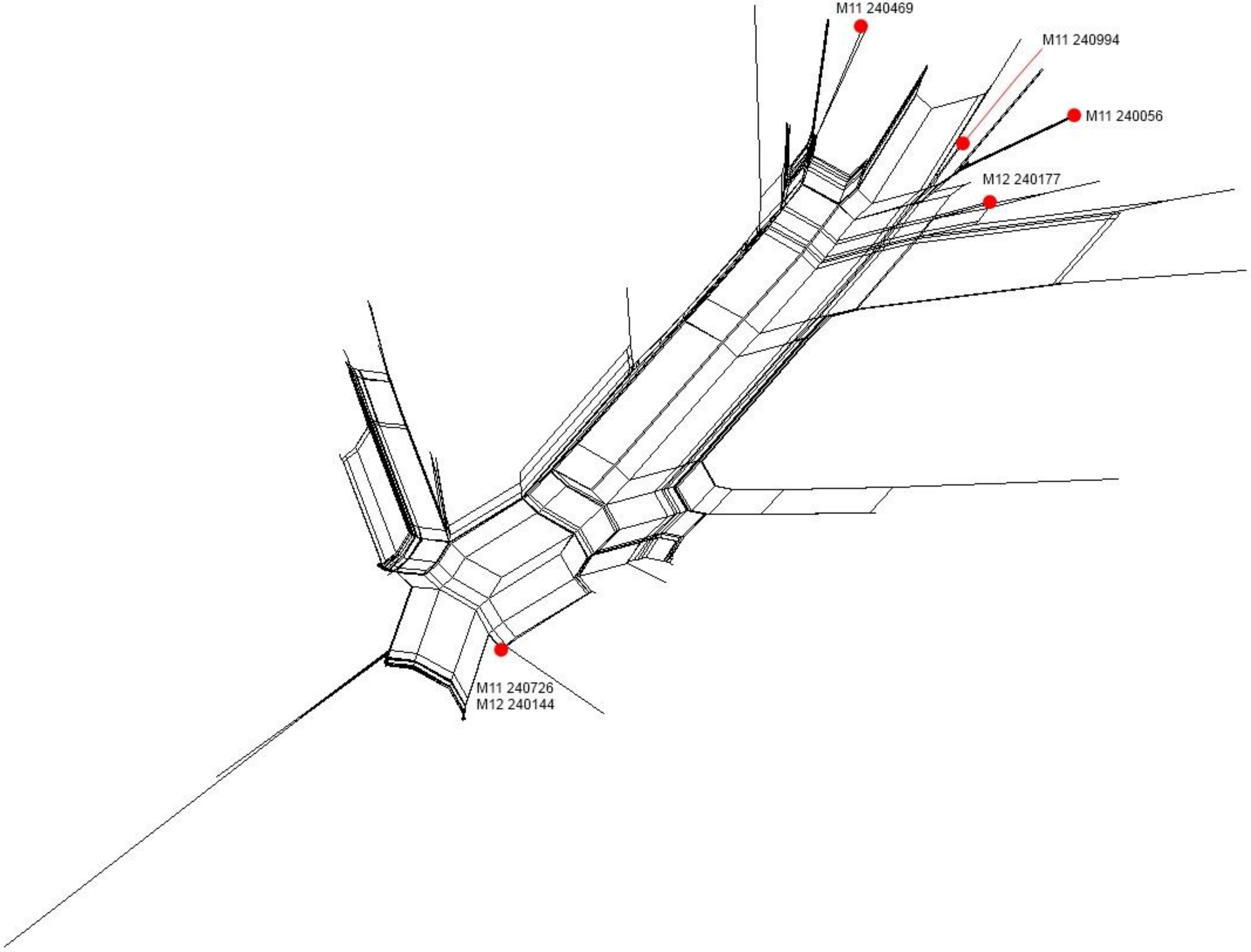
M11 240469
M11 240994
M11 240056
M12 240177
M11 240726
M12 240144

Figure S1: eBURST and SplitsTree diagrams illustrating the distribution of the validation panel isolates among the known meningococcal population diversity.

eBURST analysis was performed using all MLST allelic profile data obtained from the PubMLST database on 30/12/2013. Briefly, the eBURST program divides STs into groups based on the relatedness of the allelic variants at the seven MLST loci. For this analysis, the most stringent level was used in which a minimum of 6 identical loci is required for group definition. Single locus variants (SLVs, i.e. STs that differ at only one of the seven MLST loci) are grouped together around a 'founder ST', which possesses the most related SLVs. These are predicted to represent an ancestral ST from which all other ST variants were derived. Each group roughly correlates with established clonal complexes in which constituent STs have ≥ 4 loci in common with the ancestral ST. The eBURSTs are annotated with the STs of the validation panel isolates (highlighted in red) to illustrate their genetic diversity. The eBURSTs roughly correlate with 7 predominant clonal complexes: ST-41/44 complex (A), ST-269 complex (B), ST-32 complex (C), ST-22 and ST-23 complexes (D), ST-213 complex (E) and ST-60 complex (F). ST-10281 is a member of ST-213 complex, however, in this analysis, there is no SLV linking ST-10281 to a member of this eBURST group.

For the ST-11 complex, four of the six validation panel isolates were of ST-11. The higher resolution provided by ribosomal MLST was utilised to produce a SplitsTree diagram (G) of rMLST profile of all ST-11 complex genomes within the PubMLST database (n= 177). The isolates used in the assay validation were then highlighted to illustrate the diversity.
