# Supplementary material for: Genotypic Analysis of Meningococcal Factor H-Binding Protein from Non-Culture Clinical Specimens
Source: PLoS One. 2014 Feb 24;9(2):e89921. doi: 10.1371/journal.pone.0089921 (PMC3933679; doi:10.1371/journal.pone.0089921)
Supplement: Table S1 — N. meningitidis and N. lactamica validation isolates and clinical specimens. (PDF) [file pone.0089921.s002.pdf]

Table S1: *N. meningitidis* and *N. lactamica* validation isolates and clinical specimens

Clinical Isolates					Clinical Specimens				
**ID**	**Group**	**Sequence Type**	**Clonal Complex**	**MGL-assigned *fHbp* allele**	**ID**	**Specimen Type***	***ctrA* Ct Value****	**Specimen volume (µL) used in extraction**	**Description**
M11 240475	B	4051	Singleton	4	M11 942010	EDTA	29	100	Validation Panel Isolate/Specimen Pair
M11 240723	B	1345	Singleton	14	M11 951518	EDTA	33	10	Validation Panel Isolate/Specimen Pair
M11 240002	B	4954	Singleton	14	M11 900874	CSF	44	50	Validation Panel Isolate/Specimen Pair
M11 240323	B	9847	Singleton	13	M11 919874	EDTA	34	100	Validation Panel Isolate/Specimen Pair
M11 240402	B	9825	Singleton	No allele detected	M11 929760	CSF	33	70	Validation Panel Isolate/Specimen Pair
M11 240234	C	5238	Singleton	1	M11 913431	EDTA	27	100	Validation Panel Isolate/Specimen Pair
M12 240194	B	5151	Singleton	1	M12 910062	BLOOD	26	100	Validation Panel Isolate/Specimen Pair
M12 240120	B	10283	Singleton	19	M12 900860	BLOOD	27	100	Validation Panel Isolate/Specimen Pair
M11 240347	C	5133	ST-103 complex	19	M11 922854	EDTA	34	100	Validation Panel Isolate/Specimen Pair
M11 240056	B	11	ST-11 complex	715	M11 900926	CSF	18	100	Validation Panel Isolate/Specimen Pair
M11 240726	W	11	ST-11 complex	715	M11 951934	EDTA	27	100	Validation Panel Isolate/Specimen Pair
M11 240994	C	11	ST-11 complex	787	M11 963097	EDTA	38	100	Validation Panel Isolate/Specimen Pair
M12 240177	C	11	ST-11 complex	361	M12 908623	BLOOD	34	100	Validation Panel Isolate/Specimen Pair
M11 240469	B	10260	ST-11 complex	100	M11 941014	EDTA	23	100	Validation Panel Isolate/Specimen Pair
M12 240144	W	10284	ST-11 complex	22	M12 904562	BLOOD	32	100	Validation Panel Isolate/Specimen Pair
M11 240338	B	3737	ST-1157 complex	68	M11 921330	EDTA	26	100	Validation Panel Isolate/Specimen Pair
M11 241026	B	10268	ST-1157 complex	68	M11 964223	EDTA	31	100	Validation Panel Isolate/Specimen Pair
M11 240479	B	162	ST-162 complex	21	M11 941974	CSF	31	100	Validation Panel Isolate/Specimen Pair
M11 240045	B	858	ST-18 complex	36	M11 900891	EDTA	23	10	Validation Panel Isolate/Specimen Pair
M11 241023	B	5529	ST-18 complex	626	M11 964187	EDTA	31	100	Validation Panel Isolate/Specimen Pair
M12 240145	B	213	ST-213 complex	29	M12 904524	BLOOD	30	100	Validation Panel Isolate/Specimen Pair
M12 240092	B	3496	ST-213 complex	1	M12 898016	BLOOD	28	100	Validation Panel Isolate/Specimen Pair
M12 240208	B	9884	ST-213 complex	44	M12 911076	BLOOD	27	100	Validation Panel Isolate/Specimen Pair
M12 240107	B	10281	ST-213 complex	205	M12 899817	BLOOD	26	100	Validation Panel Isolate/Specimen Pair
M11 240349	W	22	ST-22 complex	69	M11 922561	EDTA	29	100	Validation Panel Isolate/Specimen Pair
M11 240231	W	184	ST-22 complex	16	M11 912817	EDTA	33	100	Validation Panel Isolate/Specimen Pair
M11 240029	W	1286	ST-22 complex	74	M11 900986	EDTA	28	100	Validation Panel Isolate/Specimen Pair
M11 241064	Y	3651	ST-22 complex	25	M11 969988	EDTA	33	100	Validation Panel Isolate/Specimen Pair
M11 240312	Y	23	ST-23 complex	25	M11 918182	SERUM	36	100	Validation Panel Isolate/Specimen Pair
M11 240982	Y	1655	ST-23 complex	25	M11 959902	EDTA	30	100	Validation Panel Isolate/Specimen Pair
M11 240411	Y	4183	ST-23 complex	25	M11 932904	EDTA	28	100	Validation Panel Isolate/Specimen Pair
M11 240030	B	269	ST-269 complex	15	M11 900480	EDTA	31	100	Validation Panel Isolate/Specimen Pair
M11 240368	B	269	ST-269 complex	15	M11 925218	EDTA	34	75	Validation Panel Isolate/Specimen Pair
M11 240212	B	275	ST-269 complex	19	M11 912092	EDTA	27	100	Validation Panel Isolate/Specimen Pair
M11 240775	B	283	ST-269 complex	15	M11 955585	EDTA	34	100	Validation Panel Isolate/Specimen Pair
M12 240323	C	467	ST-269 complex	15	M12 925366	BLOOD	33	100	Validation Panel Isolate/Specimen Pair
M11 240151	B	479	ST-269 complex	15	M11 906706	CSF	20	100	Validation Panel Isolate/Specimen Pair
M11 240185	B	1049	ST-269 complex	717	M11 909484	CSF	24	100	Validation Panel Isolate/Specimen Pair
M11 240484	B	1163	ST-269 complex	235	M11 943211	EDTA	33	100	Validation Panel Isolate/Specimen Pair
M12 240116	B	1195	ST-269 complex	15	M12 901115	CSF	42	95	Validation Panel Isolate/Specimen Pair
M11 240086	B	1774	ST-269 complex	149	M11 902822	EDTA	26	100	Validation Panel Isolate/Specimen Pair
M12 240216	B	4713	ST-269 complex	13	M12 911596	BLOOD	33	100	Validation Panel Isolate/Specimen Pair
M12 240303	B	5849	ST-269 complex	13	M12 921916	BLOOD	27	100	Validation Panel Isolate/Specimen Pair
M11 240334	B	6604	ST-269 complex	13	M11 920780	EDTA	28	100	Validation Panel Isolate/Specimen Pair
M11 240993	B	7226	ST-269 complex	15	M11 962537	EDTA	28	100	Validation Panel Isolate/Specimen Pair
M11 240388	B	9823	ST-269 complex	15	M11 927727	EDTA	25	100	Validation Panel Isolate/Specimen Pair
M11 240309	B	9837	ST-269 complex	19	M11 919141	EDTA	31	100	Validation Panel Isolate/Specimen Pair
M11 240243	B	9845	ST-269 complex	13	M11 913874	EDTA	30	100	Validation Panel Isolate/Specimen Pair
M11 240501	B	10263	ST-269 complex	19	M11 945434	CSF	30	50	Validation Panel Isolate/Specimen Pair
M11 240593	B	10264	ST-269 complex	15	M11 946553	EDTA	35	100	Validation Panel Isolate/Specimen Pair
M11 240189	B	8068	ST-282 complex	69	M11 909694	EDTA	26	100	Validation Panel Isolate/Specimen Pair
M11 240285	B	33	ST-32 complex	151	M11 916311	EDTA	36	100	Validation Panel Isolate/Specimen Pair
M11 240082	B	290	ST-32 complex	1	M11 902644	CLOT	25	100	Validation Panel Isolate/Specimen Pair
M11 240013	B	9890	ST-32 complex	593	M11 900970	EDTA	29	75	Validation Panel Isolate/Specimen Pair
M12 240149	B	10286	ST-32 complex	272	M12 904853	BLOOD	29	100	Validation Panel Isolate/Specimen Pair
M11 240742	B	35	ST-35 complex	708	M11 953368	EDTA	34	100	Validation Panel Isolate/Specimen Pair
M11 240060	B	2380	ST-35 complex	92	M11 901430	EDTA	27	100	Validation Panel Isolate/Specimen Pair
M11 241013	B	41	ST-41/44 complex	788	M11 963565	EDTA	31	100	Validation Panel Isolate/Specimen Pair
M12 240131	NG	41	ST-41/44 complex	4	M12 902371	BLOOD	32	100	Validation Panel Isolate/Specimen Pair
M12 240168	B	43	ST-41/44 complex	19	M12 906589	BLOOD	31	100	Validation Panel Isolate/Specimen Pair
M12 240128	B	46	ST-41/44 complex	4	M12 901848	BLOOD	29	100	Validation Panel Isolate/Specimen Pair
M11 240295	B	303	ST-41/44 complex	4	M11 917928	CSF	29	100	Validation Panel Isolate/Specimen Pair
M12 240169	NG	414	ST-41/44 complex	19	M12 906176	BLOOD	28	100	Validation Panel Isolate/Specimen Pair
M11 240119	B	437	ST-41/44 complex	30	M11 904062	EDTA	32	100	Validation Panel Isolate/Specimen Pair
M11 240088	B	485	ST-41/44 complex	4	M11 903062	EDTA	31	100	Validation Panel Isolate/Specimen Pair
M12 240296	B	571	ST-41/44 complex	19	M12 921414	CSF	27	50	Validation Panel Isolate/Specimen Pair
M11 240255	B	1090	ST-41/44 complex	4	M11 915226	EDTA	26	100	Validation Panel Isolate/Specimen Pair
M11 240193	B	1097	ST-41/44 complex	562	M11 910517	EDTA	27	100	Validation Panel Isolate/Specimen Pair
M11 241044	B	1194	ST-41/44 complex	4	M11 966768	EDTA	28	100	Validation Panel Isolate/Specimen Pair
M11 240195	B	1475	ST-41/44 complex	14	M11 910483	CSF	28	100	Validation Panel Isolate/Specimen Pair
M11 240244	B	1960	ST-41/44 complex	14	M11 913861	EDTA	26	100	Validation Panel Isolate/Specimen Pair
M11 240472	B	2009	ST-41/44 complex	24	M11 940457	EDTA	26	100	Validation Panel Isolate/Specimen Pair
M11 240145	B	2080	ST-41/44 complex	19	M11 906944	EDTA	37	100	Validation Panel Isolate/Specimen Pair
M12 240309	B	2266	ST-41/44 complex	14	M12 923425	CSF	24	90	Validation Panel Isolate/Specimen Pair
M12 240187	B	2314	ST-41/44 complex	4	M12 909491	BLOOD	36	100	Validation Panel Isolate/Specimen Pair
M11 240097	B	2799	ST-41/44 complex	4	M11 904290	CSF	22	100	Validation Panel Isolate/Specimen Pair
M11 240125	B	3818	ST-41/44 complex	4	M11 905224	CSF	33	100	Validation Panel Isolate/Specimen Pair
M11 240034	B	5861	ST-41/44 complex	73	M11 900929	CSF	33	40	Validation Panel Isolate/Specimen Pair
M11 240766	B	8054	ST-41/44 complex	14	M11 954754	EDTA	23	100	Validation Panel Isolate/Specimen Pair
M11 240506	B	8988	ST-41/44 complex	73	M11 945987	EDTA	18	100	Validation Panel Isolate/Specimen Pair
M12 240134	B	9171	ST-41/44 complex	4	M12 903101	CSF	29	100	Validation Panel Isolate/Specimen Pair
M11 240457	B	9200	ST-41/44 complex	356	M11 940244	CSF	28	100	Validation Panel Isolate/Specimen Pair
M12 240315	B	9352	ST-41/44 complex	14	M12 923786	BLOOD	37	100	Validation Panel Isolate/Specimen Pair
M11 240018	B	9820	ST-41/44 complex	4	M11 900925	EDTA	26	100	Validation Panel Isolate/Specimen Pair
M11 240431	B	9827	ST-41/44 complex	19	M11 934622	EDTA	23	100	Validation Panel Isolate/Specimen Pair
M11 240059	B	9832	ST-41/44 complex	4	M11 902886	EDTA	32	100	Validation Panel Isolate/Specimen Pair
M11 240728	B	9889	ST-41/44 complex	713	M11 951931	EDTA	27	100	Validation Panel Isolate/Specimen Pair
M11 240311	B	9896	ST-41/44 complex	4	M11 919007	EDTA	30	100	Validation Panel Isolate/Specimen Pair
M11 240170	B	10139	ST-41/44 complex	281	M11 906901	EDTA	22	100	Validation Panel Isolate/Specimen Pair
M11 240988	B	461	ST-461 complex	71	M11 962849	EDTA	23	100	Validation Panel Isolate/Specimen Pair
M11 240262	A	4789	ST-5 complex	719	M11 915746	EDTA	30	100	Validation Panel Isolate/Specimen Pair
M11 240440	B	60	ST-60 complex	13	M11 936564	BLOOD	29	100	Validation Panel Isolate/Specimen Pair
M11 240394	B	5103	ST-60 complex	13	M11 928895	EDTA	24	80	Validation Panel Isolate/Specimen Pair
M11 240167	B	9327	ST-60 complex	13	M11 907243	EDTA	25	100	Validation Panel Isolate/Specimen Pair
M10 240825	B	9818	ST-60 complex	13	M11 900880	SERUM	39	100	Validation Panel Isolate/Specimen Pair
M12 240222	B	10292	ST-60 complex	13	M12 912364	BLOOD	28	100	Validation Panel Isolate/Specimen Pair

Clinical Isolates					Clinical Specimens				
ID	Group	Sequence Type	Clonal Complex	MGL-assigned *fHbp* allele	ID	Specimen Type*	*ctrA* Ct Value**	Specimen volume (µL) used in extraction	Description
M08 0240297	B	32	ST-32 complex	1	n/a	n/a	n/a	n/a	Analytical Sensitivity/Primer Optimisation Isolate
M07 0240954	C	491	ST-11 complex	10	n/a	n/a	n/a	n/a	Analytical Sensitivity Isolate
M07 0241036	B	269	ST-269 complex	15	n/a	n/a	n/a	n/a	Analytical Sensitivity Isolate
M07 0241073	B	162	ST-162 complex	21	n/a	n/a	n/a	n/a	Analytical Sensitivity Isolate
M07 0240725	B	2080	ST-41/44 complex	348	n/a	n/a	n/a	n/a	Analytical Sensitivity/Primer Optimisation Isolate
M08 0240113	B	213	ST-213 complex	30	n/a	n/a	n/a	n/a	Analytical Sensitivity/Primer Optimisation Isolate
M08 0240032	B	461	ST-461 complex	71	n/a	n/a	n/a	n/a	Analytical Sensitivity Isolate
M98 250306	n/a	640	ST-640 complex	n/a	n/a	n/a	n/a	n/a	*N. lactamica* Isolate
M00 240031	n/a	624	ST-624 complex	n/a	n/a	n/a	n/a	n/a	*N. lactamica* Isolate
M03 240246	n/a	609	ST-613 complex (putative)	n/a	n/a	n/a	n/a	n/a	*N. lactamica* Isolate
M98 250219	n/a	4192	Cluster 4192	n/a	n/a	n/a	n/a	n/a	*N. lactamica* Isolate
M03 241253	n/a	9417	ST-613 complex	n/a	n/a	n/a	n/a	n/a	*N. lactamica* Isolate
M99 242475	n/a	1494	ST-1494 complex	n/a	n/a	n/a	n/a	n/a	*N. lactamica* Isolate

***EDTA- Ethylenediaminetetraacetic Acid-Treated Blood Sample, BLOOD- Blood Sample, CSF- Cerebrospinal Fluid Sample, SERUM- Serum Sample, CLOT- Clotted Blood Sample.**

**** Derived using *ctrA* -directed real time PCR. See Methods: Analytical Sensitivity.**
