# Supplementary material for: Genotypic Analysis of Meningococcal Factor H-Binding Protein from Non-Culture Clinical Specimens
Source: PLoS One. 2014 Feb 24;9(2):e89921. doi: 10.1371/journal.pone.0089921 (PMC3933679; doi:10.1371/journal.pone.0089921)
Supplement: Table S2 — Isolates from which fHbp and flanking sequences were used to assess primer site conservation and identify putative primer candidates. (PDF) [file pone.0089921.s003.pdf]

Table S2: Isolates from which *fHbp* and flanking sequences were used to assess primer site conservation and identify putative primer candidates.

No.	Isolate	No.	Isolate	No.	Isolate	No.	Isolate	No.	Isolate	No.	Isolate	No.	Isolate	No.	Isolate	No.	Isolate	No.	Isolate	No.	Isolate
1	M10 240473	43	M10 240636	85	M11 240023	127	M11 240068	169	M11 240211	211	M11 240335	253	M11 240453	295	M11 240594	337	M11 241040	379	M12 240254	421	M12 240326
2	M10 240474	44	M10 240638	86	M11 240024	128	M11 240069	170	M11 240212	212	M11 240337	254	M11 240456	296	M11 240595	338	M11 241042	380	M12 240255	422	M12 240328
3	M10 240478	45	M10 240640	87	M11 240025	129	M11 240070	171	M11 240214	213	M11 240338	255	M11 240457	297	M11 240597	339	M11 241043	381	M12 240257	423	M12 240329
4	M10 240480	46	M10 240641	88	M11 240026	130	M11 240071	172	M11 240215	214	M11 240339	256	M11 240458	298	M11 240598	340	M11 241044	382	M12 240261	424	M12 240330
5	M10 240481	47	M10 240642	89	M11 240027	131	M11 240072	173	M11 240216	215	M11 240344	257	M11 240459	299	M11 240599	341	M11 241046	383	M12 240263	425	M12 240332
6	M10 240482	48	M10 240643	90	M11 240028	132	M11 240073	174	M11 240227	216	M11 240352	258	M11 240461	300	M11 240710	342	M11 241047	384	M12 240264	426	M12 240333
7	M10 240484	49	M10 240649	91	M11 240029	133	M11 240074	175	M11 240231	217	M11 240353	259	M11 240463	301	M11 240712	343	M11 241048	385	M12 240267	427	M12 240334
8	M10 240507	50	M10 240651	92	M11 240030	134	M11 240100	176	M11 240233	218	M11 240358	260	M11 240465	302	M11 240713	344	M11 241050	386	M12 240272	428	M12 240335
9	M10 240508	51	M10 240652	93	M11 240031	135	M11 240111	177	M11 240236	219	M11 240360	261	M11 240466	303	M11 240716	345	M11 241051	387	M12 240273	429	alpha14
10	M10 240511	52	M10 240659	94	M11 240032	136	M11 240113	178	M11 240237	220	M11 240363	262	M11 240467	304	M11 240717	346	M11 241054	388	M12 240274	430	G2136
11	M10 240514	53	M10 240661	95	M11 240034	137	M11 240116	179	M11 240243	221	M11 240401	263	M11 240469	305	M11 240719	347	M11 241055	389	M12 240277	431	NZ-05/33
12	M10 240520	54	M10 240665	96	M11 240035	138	M11 240117	180	M11 240246	222	M11 240403	264	M11 240470	306	M11 240721	348	M11 241057	390	M12 240284	432	Z2491
13	M10 240528	55	M10 240666	97	M11 240036	139	M11 240118	181	M11 240247	223	M11 240405	265	M11 240471	307	M11 240723	349	M11 241058	391	M12 240287	433	MC58
14	M10 240530	56	M10 240668	98	M11 240037	140	M11 240119	182	M11 240249	224	M11 240406	266	M11 240472	308	M11 240724	350	M11 241059	392	M12 240288	434	FAM18
15	M10 240531	57	M10 240671	99	M11 240038	141	M11 240122	183	M11 240258	225	M11 240409	267	M11 240473	309	M11 240725	351	M11 241060	393	M12 240289	435	53442
16	M10 240534	58	M10 240675	100	M11 240039	142	M11 240123	184	M11 240261	226	M11 240412	268	M11 240474	310	M11 240726	352	M11 241061	394	M12 240290	436	ATCC 13091
17	M10 240536	59	M10 240676	101	M11 240040	143	M11 240126	185	M11 240263	227	M11 240413	269	M11 240475	311	M11 240727	353	M11 241063	395	M12 240291	437	8013
18	M10 240540	60	M10 240677	102	M11 240041	144	M11 240128	186	M11 240266	228	M11 240414	270	M11 240476	312	M11 240728	354	M11 241064	396	M12 240293	438	alpha710
19	M10 240546	61	M10 240684	103	M11 240042	145	M11 240129	187	M11 240277	229	M11 240417	271	M11 240477	313	M11 240731	355	M11 241065	397	M12 240294	439	WUE 2594
20	M10 240547	62	M10 240685	104	M11 240043	146	M11 240131	188	M11 240278	230	M11 240420	272	M11 240479	314	M11 240733	356	M11 241067	398	M12 240296	440	M01-240149
21	M10 240550	63	M10 240687	105	M11 240044	147	M11 240134	189	M11 240280	231	M11 240422	273	M11 240480	315	M11 240734	357	M11 241068	399	M12 240299	441	M04-240196
22	M10 240553	64	M10 240693	106	M11 240045	148	M11 240137	190	M11 240284	232	M11 240424	274	M11 240484	316	M11 240735	358	M11 241069	400	M12 240300	442	H44/76
23	M10 240566	65	M10 240694	107	M11 240046	149	M11 240139	191	M11 240287	233	M11 240425	275	M11 240485	317	M11 240736	359	M11 241072	401	M12 240301	443	M01-240355
24	M10 240572	66	M10 240698	108	M11 240047	150	M11 240145	192	M11 240290	234	M11 240427	276	M11 240486	318	M11 240737	360	M11 241073	402	M12 240302	L1	005-12
25	M10 240579	67	M10 240746	109	M11 240048	151	M11 240146	193	M11 240294	235	M11 240428	277	M11 240487	319	M11 240738	361	M11 241074	403	M12 240303	L2	Y92-1009
26	M10 240580	68	M10 240747	110	M11 240050	152	M11 240147	194	M11 240297	236	M11 240430	278	M11 240488	320	M11 240740	362	M11 241075	404	M12 240305	L3	028-12
27	M10 240583	69	M10 240748	111	M11 240052	153	M11 240157	195	M11 240298	237	M11 240431	279	M11 240489	321	M11 240741	363	M11 241076	405	M12 240306	L4	030-24
28	M10 240587	70	M10 240749	112	M11 240053	154	M11 240163	196	M11 240302	238	M11 240434	280	M11 240491	322	M11 240742	364	M11 241077	406	M12 240307	L5	039-03
29	M10 240590	71	M10 240750	113	M11 240054	155	M11 240166	197	M11 240303	239	M11 240435	281	M11 240492	323	M11 240743	365	M11 241078	407	M12 240308	L6	049-12
30	M10 240591	72	M10 240751	114	M11 240055	156	M11 240167	198	M11 240304	240	M11 240436	282	M11 240493	324	M11 240745	366	M12 240000	408	M12 240309	L7	8206
31	M10 240602	73	M10 240752	115	M11 240056	157	M11 240168	199	M11 240305	241	M11 240437	283	M11 240494	325	M11 240749	367	M12 240001	409	M12 240310	L8	016-24
32	M10 240606	74	M10 240753	116	M11 240057	158	M11 240174	200	M11 240309	242	M11 240440	284	M11 240495	326	M11 240750	368	M12 240002	410	M12 240314	L9	ATCC 23970
33	M10 240607	75	M10 240754	117	M11 240058	159	M11 240176	201	M11 240312	243	M11 240441	285	M11 240497	327	M11 240984	369	M12 240003	411	M12 240315	L10	004-12
34	M10 240613	76	M10 240756	118	M11 240059	160	M11 240181	202	M11 240313	244	M11 240442	286	M11 240501	328	M11 241028	370	M12 240156	412	M12 240317	L11	012-12
35	M10 240614	77	M10 240759	119	M11 240060	161	M11 240183	203	M11 240314	245	M11 240443	287	M11 240502	329	M11 241031	371	M12 240160	413	M12 240318	L12	017-02
36	M10 240616	78	M10 240761	120	M11 240061	162	M11 240189	204	M11 240315	246	M11 240445	288	M11 240506	330	M11 241032	372	M12 240245	414	M12 240319	L13	020-06
37	M10 240618	79	M10 240762	121	M11 240062	163	M11 240192	205	M11 240316	247	M11 240446	289	M11 240507	331	M11 241033	373	M12 240248	415	M12 240320	L14	NL-Y92-1009-1
38	M10 240622	80	M10 240763	122	M11 240063	164	M11 240193	206	M11 240319	248	M11 240447	290	M11 240508	332	M11 241034	374	M12 240249	416	M12 240321	L15	NL-Y92-1009-2
39	M10 240624	81	M11 240018	123	M11 240064	165	M11 240206	207	M11 240322	249	M11 240448	291	M11 240509	333	M11 241035	375	M12 240250	417	M12 240322	L16	224
40	M10 240626	82	M11 240019	124	M11 240065	166	M11 240207	208	M11 240323	250	M11 240450	292	M11 240510	334	M11 241036	376	M12 240251	418	M12 240323	n/a	n/a
41	M10 240632	83	M11 240021	125	M11 240066	167	M11 240209	209	M11 240330	251	M11 240451	293	M11 240592	335	M11 241037	377	M12 240252	419	M12 240324	n/a	n/a
42	M10 240633	84	M11 240022	126	M11 240067	168	M11 240210	210	M11 240333	252	M11 240452	294	M11 240593	336	M11 241039	378	M12 240253	420	M12 240325	n/a	n/a

DNA sequences from all isolates above were used to assess primer-site conservation. Shaded isolates were also used to identify new potential primer-sites.

Numbers of *N. lactamica* isolates are prefixed with 'L' .
