# Supplementary material for: Genotypic Analysis of Meningococcal Factor H-Binding Protein from Non-Culture Clinical Specimens
Source: PLoS One. 2014 Feb 24;9(2):e89921. doi: 10.1371/journal.pone.0089921 (PMC3933679; doi:10.1371/journal.pone.0089921)
Supplement: Table S3 — Results of PCR and sequencing of validation panel isolate/specimen pairs. (PDF) [file pone.0089921.s004.pdf]

Table S3: Results of PCR and sequencing of validation panel isolate/specimen pairs.

Clinical Specimen Results (Nested PCR)							Clinical Isolate Results (Individual PCR Rounds)				
**Specimen**	**Run 1**	**Run 2**	**Run 3**	**Run 4***	***fHbp* Allele**	**fHbp Peptide**	**Isolate**	**Round One Product**	**Round Two Product**	**MGL-assigned *fHbp* Allele**	**Sequenced *fHbp* Allele**
M11 900480	+				15	15	M11 240030	+	+	15	15
M11 900874	-	-	-	-	n/a	n/a	M11 240002	+	+	14	14
M11 900880	-	-	-	-	n/a	n/a	M10 240825	+	+	13	13
M11 900891	+				36	37	M11 240045	+	+	36	36
M11 900925	+				4	4	M11 240018	+	+	4	4
M11 900926	+				715	606	M11 240056	+	+	715	715
M11 900929	+				73	86	M11 240034	+	+	73	73
M11 900970	+				593	510	M11 240013	+	+	593	593
M11 900986	+				74	16	M11 240029	+	+	74	74
M11 901430	+				92	5	M11 240060	+	+	92	92
M11 902644	+				1	1	M11 240082	+	+	1	1
M11 902822	+				149	143	M11 240086	+	+	149	149
M11 902886	+				4	4	M11 240059	+	+	4	4
M11 903062	+				4	4	M11 240088	+	+	4	4
M11 904062	-	-	+		30	30	M11 240119	+	+	30	30
M11 904290	+				4	4	M11 240097	+	+	4	4
M11 905224	+				4	4	M11 240125	+	+	4	4
M11 906706	+				15	15	M11 240151	+	+	15	15
M11 906901	+				281	14	M11 240170	+	+	281	281
M11 906944	-	-	-	-	n/a	n/a	M11 240145	+	+	19	19
M11 907243	+				13	13	M11 240167	+	+	13	13
M11 909484	+				717	15	M11 240185	+	+	717	717
M11 909694	+				69	89	M11 240189	+	-	69	n/a
M11 910483	+				14	14	M11 240195	+	+	14	14
M11 910517	+				562	486	M11 240193	+	+	562	562
M11 912092	+				19	19	M11 240212	+	+	19	19
M11 912817	+				16	16	M11 240231	+	+	16	16
M11 913431	-	+			1	1	M11 240234	+	+	1	1
M11 966768	+				4	4	M11 241044	+	+	4	4
M11 913861	+				14	14	M11 240244	+	+	14	14
M11 913874	+				13	13	M11 240243	+	+	13	13
M11 915226	+				4	4	M11 240255	+	+	4	4
M11 915746	+				719	94	M11 240262	+	+	719	719
M11 916311	+				151	144	M11 240285	+	+	151	151
M11 917928	+				4	4	M11 240295	+	+	4	4
M11 918182	-	-	-	-	n/a	n/a	M11 240312	+	+	25	25
M11 919007	+				4	4	M11 240311	+	+	4	4
M11 919141	+				19	19	M11 240309	+	+	19	19
M11 919874	+				13	13	M11 240323	+	+	13	13
M11 920780	+				13	13	M11 240334	+	+	13	13
M11 921330	+				68	13	M11 240338	+	+	68	68
M11 922561	+				69	84	M11 240349	+	+	69	69
M11 922854	-	+			19	19	M11 240347	+	+	19	19
M11 925218	+				15	15	M11 240368	+	+	15	15
M11 927727	+				15	15	M11 240388	+	+	15	15
M11 928895	+				13	13	M11 240394	+	+	13	13
M11 929760	-	-	-	+	NLA18150	NLA18150	M11 240402	+	+	No Allele in MRF	No Trace Obtained
M11 932904	+				25	25	M11 240411	+	+	25	25
M11 934622	+				19	19	M11 240431	+	+	19	19
M11 936564	+				13	13	M11 240440	+	+	13	13
M11 940244	+				356	302	M11 240457	+	+	356	356
M11 940457	+				24	24	M11 240472	+	+	24	24
M11 941014	+				100	100	M11 240469	+	+	100	100
M11 941974	-	+			21	21	M11 240479	+	+	21	21
M11 942010	+				4	4	M11 240475	+	+	4	4
M11 943211	+				235	215	M11 240484	+	+	235	235

Clinical Specimen Results (Nested PCR)							Clinical Isolate Results (Individual PCR Rounds)				
**Specimen**	**Run 1**	**Run 2**	**Run 3**	**Run 4***	***fHbp* Allele**	**fHbp Peptide**	**Isolate**	**Round One Product**	**Round Two Product**	**MGL-assigned *fHbp* Allele**	**Sequenced *fHbp* Allele**
M11 945434	+				19	19	M11 240501	+	+	19	19
M11 945987	+				73	86	M11 240506	+	+	73	73
M11 946553	+				15	15	M11 240593	+	+	15	15
M11 951518	+				14	14	M11 240723	+	+	14	14
M11 951931	+				713	8	M11 240728	+	+	713	713
M11 951934	+				22	22	M11 240726	+	+	22	22
M11 953368	-	-	+		708	708	M11 240742	+	+	708	708
M11 954754	+				14	14	M11 240766	+	+	14	14
M11 955585	+				15	15	M11 240775	+	+	15	15
M11 959902	+				25	25	M11 240982	+	+	25	25
M11 962537	+				15	15	M11 240993	+	+	15	15
M11 962849	+				71	47	M11 240988	+	+	71	71
M11 963097	+				787	650	M11 240994	+	+	787	787
M11 963565	+				788	651	M11 241013	+	+	788	788
M11 964187	+				626	539	M11 241023	+	+	626	626
M11 964223	+				68	13	M11 241026	+	+	68	68
M11 969988	+				25	25	M11 241064	+	+	25	25
M12 898016	+				1	1	M12 240092	+	+	1	1
M12 899817	+				205	187	M12 240107	+	+	205	205
M12 900860	+				19	19	M12 240120	+	+	19	19
M12 901115	+				15	15	M12 240116	+	+	15	15
M12 901848	+				4	4	M12 240128	+	+	4	4
M12 902371	+				4	4	M12 240131	+	+	4	4
M12 903101	-	-	-	+	4	4	M12 240134	+	+	4	4
M12 904524	+				29	29	M12 240145	+	+	29	29
M12 904562	+				22	22	M12 240144	+	+	22	22
M12 904853	+				272	224	M12 240149	+	+	272	272
M12 906176	+				19	19	M12 240169	+	+	19	19
M12 906589	+				19	19	M12 240168	+	+	19	19
M12 908623	+				361	306	M12 240177	+	+	361	361
M12 909491	-	-	-	-	n/a	n/a	M12 240187	+	+	4	4
M12 910062	+				1	1	M12 240194	+	+	1	1
M12 911076	+				44	59	M12 240208	+	+	44	44
M12 911596	-	+			13	13	M12 240216	+	+	13	13
M12 912364	+				13	13	M12 240222	+	+	13	13
M12 921414	+				19	19	M12 240296	+	+	19	19
M12 921916	+				13	13	M12 240303	+	+	13	13
M12 923425	+				14	14	M12 240309	+	+	14	14
M12 923786	+				14	14	M12 240315	+	+	14	14
M12 925366	+				15	15	M12 240323	+	+	15	15

* Samples tested at a 20µl /100µl PCR round one extract/reaction volume ratio. 45 and 35 PCR cycles were used for round one and round two, respectively.
