# Supplementary material for: Genotypic Analysis of Meningococcal Factor H-Binding Protein from Non-Culture Clinical Specimens
Source: PLoS One. 2014 Feb 24;9(2):e89921. doi: 10.1371/journal.pone.0089921 (PMC3933679; doi:10.1371/journal.pone.0089921)
Supplement: Method S1 — Assay Development. (PDF) [file pone.0089921.s005.pdf]

Supporting Information: Assay Development

Assessment of Primer Suitability

All primers used are listed in Table 2. The primers were primarily chosen based on the location of the complementary sequence in relation to *fHbp*. Appropriate melting temperatures (Tm, 55-60°C) and percentage GC content (%CG, 40-60%) were confirmed. To assess the level of conservation, DNA sequences of *fHbp* and flanking regions from a range of meningococci (n= 443) were retrieved from the PubMLST database (Table S2). These were aligned and compared to primer sequences using BioEdit DNA alignment software. The specificity of potential primer candidates was assessed by searching for complementary targets using the NCBI nucleotide BLAST tool (http://blast.ncbi.nlm.nih.gov/Blast.cgi). Testing for non-specific binding within the target region was performed following deletion of the corresponding primers-sites from alignments using Sequencher v4.7 (Gene Codes Corporation, Ann Arbor, MI USA).

Existing Primers

1869-2F and *1871Ralt* have been used previously (29). The complementary binding sites are located within the genes flanking *fHbp* and therefore show a good level of conservation. Three of the four sequencing primers adopted for the assay were initially published by Jacobsson et al. (30). Potential non-specific binding of the fourth primer of this set, *gna1870R*, to alternative sites resulted in poor quality sequence traces and prompted its replacement with *fHbpseqR2*.

Design of New Primers

fHbpRd2F and *fHbpRd2R* were designed for use in the second PCR round. *fHbpseqR2* was designed to replace *gna1870R* as an outer reverse sequencing primer. All three primers are located within genes flanking *fHbp*.

fHbpRd2R contains a degenerate base at position 18 (5 ’to 3’) and *fHbpseqR2* contains two degenerate sites at positions 9 and 15 (5’ to 3’). These were included to improve the effectiveness of the primers against diverse strains.

To identify suitable primer-sites, DNA sequences in the flanking regions of *fHbp* from 15 isolates were obtained from GenBank (Table S2). Accession numbers: NC_013016 (alpha14), NC_017513 (G2136), NC_017518 (NZ-05/33), NC_003116 (Z2491), NC_003112 (MC58); NC_008767 (FAM18), NC_010120 (053442), NZ_AEEF00000000 (ATCC 13091)

NC_017501 (8013), NC_017505 (alpha710), NC_017512 (WUE 2594), NC_017514 (M01-240149), NC_017515 (M04-240196), NC_017516 (H44/76), NC_017517 (M01-240355). The consensus of these sequences was entered into Integrated DNA Technologies (IDT) 'Primer Quest' online software (https://eu.idtdna.com/PrimerQuest/Home/Index) to produce a list of putative primer candidates. These primers were then checked for conservation. Suitably conserved primers were checked for %GC, Tm and specificity to target site (See 'Assessment of Primer Suitability’).

Optimisation of Newly Designed Primers

Primer optimisation was carried out empirically through the alteration of annealing temperature (Ta), $MgCl_2$ concentration and cycle repeat number. DNA extracted from 3 invasive meningococcal isolates, each expressing fHbp of one of the three variant groups, was used for optimisation (Table S1). The optimum conditions were identified through visual assessment of products on an agarose gel.
